# TMPRSS13 promotes cell survival, invasion, and resistance to drug-induced apoptosis in colorectal cancer

**DOI:** 10.1038/s41598-020-70636-4

**Published:** 2020-08-17

**Authors:** Fausto A. Varela, Victoria L. Foust, Thomas E. Hyland, Kimberley E. Sala-Hamrick, Jacob R. Mackinder, Carly E. Martin, Andrew S. Murray, Sokol V. Todi, Karin List

**Affiliations:** 1 Department of Pharmacology, Wayne State University School of Medicine, Detroit, 48201 MI USA; 2 Department of Oncology, Wayne State University School of Medicine, Detroit, 48201 MI USA; 3 Department of Neurology, Wayne State University School of Medicine, Detroit, 48201 MI USA

**Keywords:** Cancer, Cell biology

## Abstract

Cancer progression is often accompanied by increased levels of extracellular proteases capable of remodeling the extracellular matrix and promoting pro-cancerous signaling pathways by activating growth factors and receptors. The type II transmembrane serine protease (TTSP) family encompasses several proteases that play critical roles in cancer progression; however, the expression or function of the TTSP TMPRSS13 in carcinogenesis has not been examined. In the present study, we found TMPRSS13 to be differentially expressed at both the transcript and protein levels in human colorectal cancer (CRC). Immunohistochemical analyses revealed consistent high expression of TMPRSS13 protein on the cancer cell surface in CRC patient samples; in contrast, the majority of normal colon samples displayed no detectable expression. On a functional level, TMPRSS13 silencing in CRC cell lines increased apoptosis and impaired invasive potential. Importantly, transgenic overexpression of TMPRSS13 in CRC cell lines increased tolerance to apoptosis-inducing agents, including paclitaxel and HA14-1. Conversely, TMPRSS13 silencing rendered CRC cells more sensitive to these agents. Together, our findings suggest that TMPRSS13 plays an important role in CRC cell survival and in promoting resistance to drug-induced apoptosis; we also identify TMPRSS13 as a potential new target for monotherapy or combination therapy with established chemotherapeutics to improve treatment outcomes in CRC patients.

## Introduction

According to the American Cancer Society, colorectal cancer (CRC) is the third most common cancer and the second leading cause of cancer-related deaths in both genders in the United States^1^. Early diagnosis of disease can lead to successful treatment through surgical interventions, although the prognosis for advanced and metastatic CRC is poor due to limited medical treatment options. Fluorouracil (5-FU) and its pro-drug form capecitabine are currently the most frequently used agents, alone or in combination with drugs such as oxaliplatin and irinotecan^2–4^. Although targeted therapies have been successful in the treatment of some types of cancers, such as breast cancer, they have limited efficacy in adjuvant treatment of colorectal cancer (i.e., cetuximab, panitumumab, bevacizumab, ramucirumab, ziv-aflibercept, and regorafenib) and add relatively small survival benefits for those with advanced disease^5–7^. Therefore, there is an urgent need to develop novel drug regimens for patients suffering from advanced CRC. To this end, understanding the molecular mechanisms driving CRC represents a critical step toward the development of novel targeted therapeutics for this particularly deadly type of cancer.

Proteolysis is a tightly regulated process under normal physiological conditions and proteolytic dysregulation constitutes both a hallmark of cancer and a contributing factor. The type II transmembrane serine protease (TTSP) subfamily is a relatively new classification of membrane-anchored serine proteases; many TTSPs play key roles in processes exploited by cancer, such as tissue remodeling, cellular migration and invasion, and metastasis^8–13^. Cancer-focused studies using cell culture and animal models have identified the pro-oncogenic properties of several TTSPs^14–18^.

TMPRSS13 (transmembrane protease, serine 13; also known as mosaic serine protease large-form, or MSPL) is expressed in several epithelial tissues, such as the epithelia of the oral cavity, esophagus, bladder, stomach, and skin^19,20^. TMPRSS13-deficiency causes abnormal development of the epidermal stratum corneum in newborn mice and leads to mildly compromised barrier function, detectable as an increased rate of trans-epidermal fluid loss in neonates^19^. This phenotype is transient, with the aberrant epidermal stratum corneum of the newborn mice not observed in adults^19^. Long-term studies (up to 12 months of age) show that TMPRSS13-deficient mice are outwardly healthy and present no detectable histological tissue abnormalities. TMPRSS13 is also expressed in mouse and human respiratory epithelium and several studies show a role for TMPRSS13 in influenza infection by proteolytically modifying the viral protein hemagglutinin, which is necessary for virus infectivity^21–24^. To date, two mammalian substrates, the pro-form of hepatocyte growth factor (HGF) and the epithelial sodium channel (ENaC), have been shown to be activated by TMPRSS13 in vitro^25,26^.

In this study, we identify TMPRSS13 as a differentially expressed TTSP in human CRC. Functionally, we present a role for TMPRSS13 in CRC cell survival, invasiveness, and resistance to apoptosis-inducing agents.

## Results

### TMPRSS13 is upregulated in human colorectal cancer

**Figure 1:**
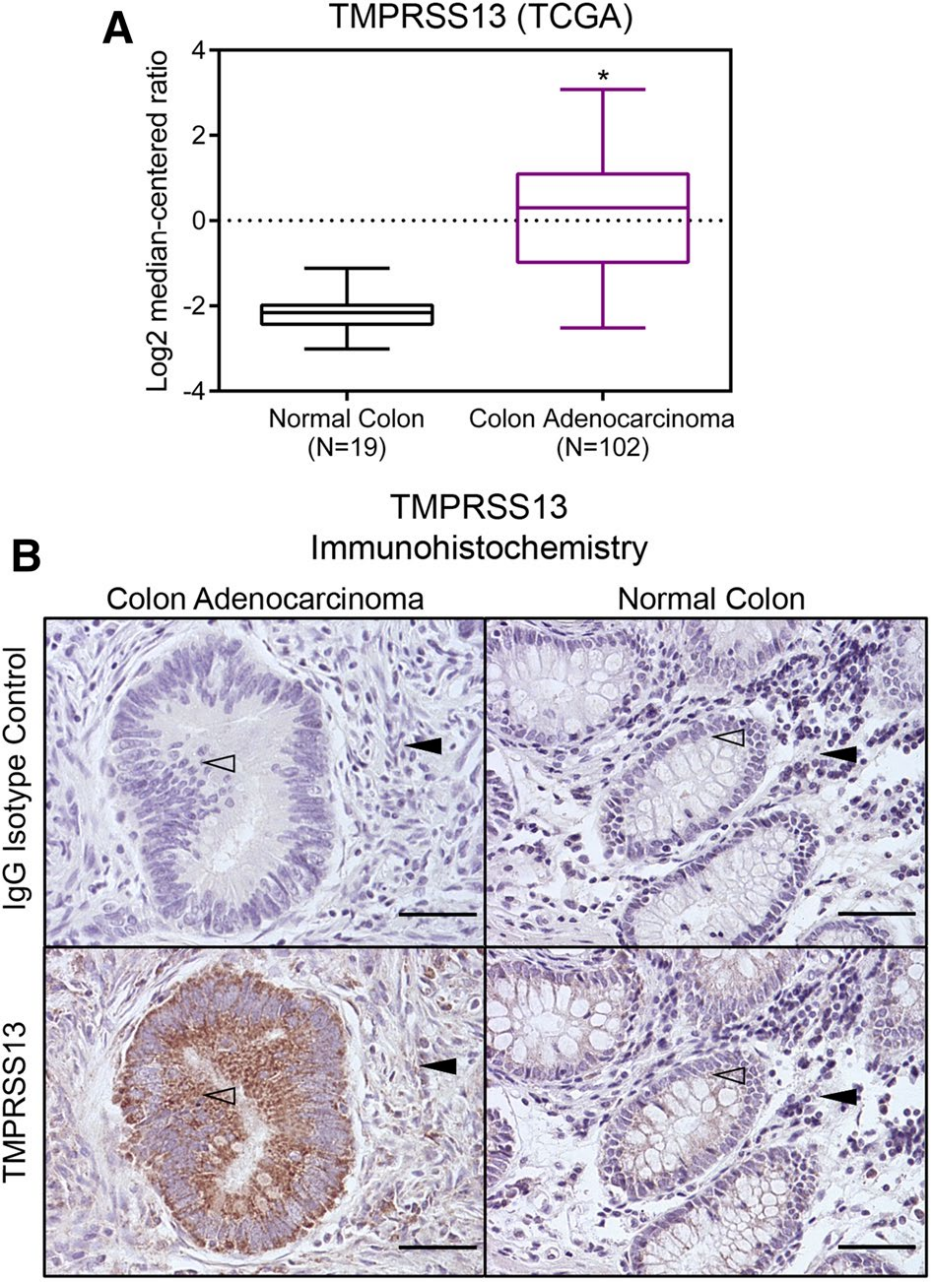
TMPRSS13 transcript and protein are upregulated in human colorectal cancer. (**A**) Box-and-whisker plot showing TMPRSS13 mRNA expression data in normal and colon adenocarcinoma tissue samples (The Cancer Genome Atlas (TCGA))^27,71^. TMPRSS13 gene expression values in normal colon (N = 19, black) and colon adenocarcinoma tissue (N = 102, purple) are shown. Box-and-whisker plots represent interquartile ranges with the median indicated as the horizontal line inside boxes (*p = 2.96 × 10^−26^, fold change = 4.918). The results are based upon data generated by the TCGA Research Network: https://www.cancer.gov/tcga. (**B**) Representative samples from tissue array IHC analysis of TMPRSS13 protein expression in normal human colon (right panels) and colon adenocarcinoma (left panels) samples. Primary, rabbit anti-TMPRSS13 antibody was substituted with non-immune rabbit IgG in serial sections of all samples and no significant staining was observed (upper panels). Open arrowheads indicate epithelial cells with undetectable or weak TMPRSS13 staining in normal colon (lower right panel) compared to strong TMPRSS13 staining in epithelial cells of a grade II colon adenocarcinoma (lower left panel). The lamina propria is indicated with black arrowheads. Scale bars = 50 µm.

As part of an ongoing effort to determine the expression and function of the TTSP family in healthy colon tissue and CRC, we performed a systematic expression analysis of TTSPs in cancer through in silico data mining using the Oncomine™ microarray database. TMPRSS13 transcripts were found to be significantly upregulated in human colon adenocarcinomas compared to normal human colon^27^ (Fig. 1A). TMPRSS13 expression in the normal colon has previously been reported to be low or undetectable in both human and mouse tissue^19,23,28^. To confirm the elevated levels of TMPRSS13 at the protein level in human CRC, we performed an immunohistochemical (IHC) analysis on human colon tissue arrays. Low protein expression levels of TMPRSS13 were detected in colon crypt epithelial cells in some normal samples (~ 2%) (Fig. 1B, lower right panel); however, the majority of normal colon samples displayed no detectable expression. Strong expression of TMPRSS13 was detected in epithelial-derived colon adenocarcinoma (Fig. 1B, lower left panel). TMPRSS13 protein localized on the cell surface of the epithelial cells and no nuclear staining was observed. This cell surface localization is in agreement with the expected membrane-anchored structure of TMPRSS13. No significant staining was observed when primary antibodies were substituted with non-immune rabbit IgG in serial sections of all samples (Fig. 1B, upper panels). Antibody specificity was verified using IHC of known low-expressing versus high-expressing normal human tissues and western blot analysis of TMPRSS13-expressing HEK293T cells (Supplementary Fig. 5).

**Figure 2:**
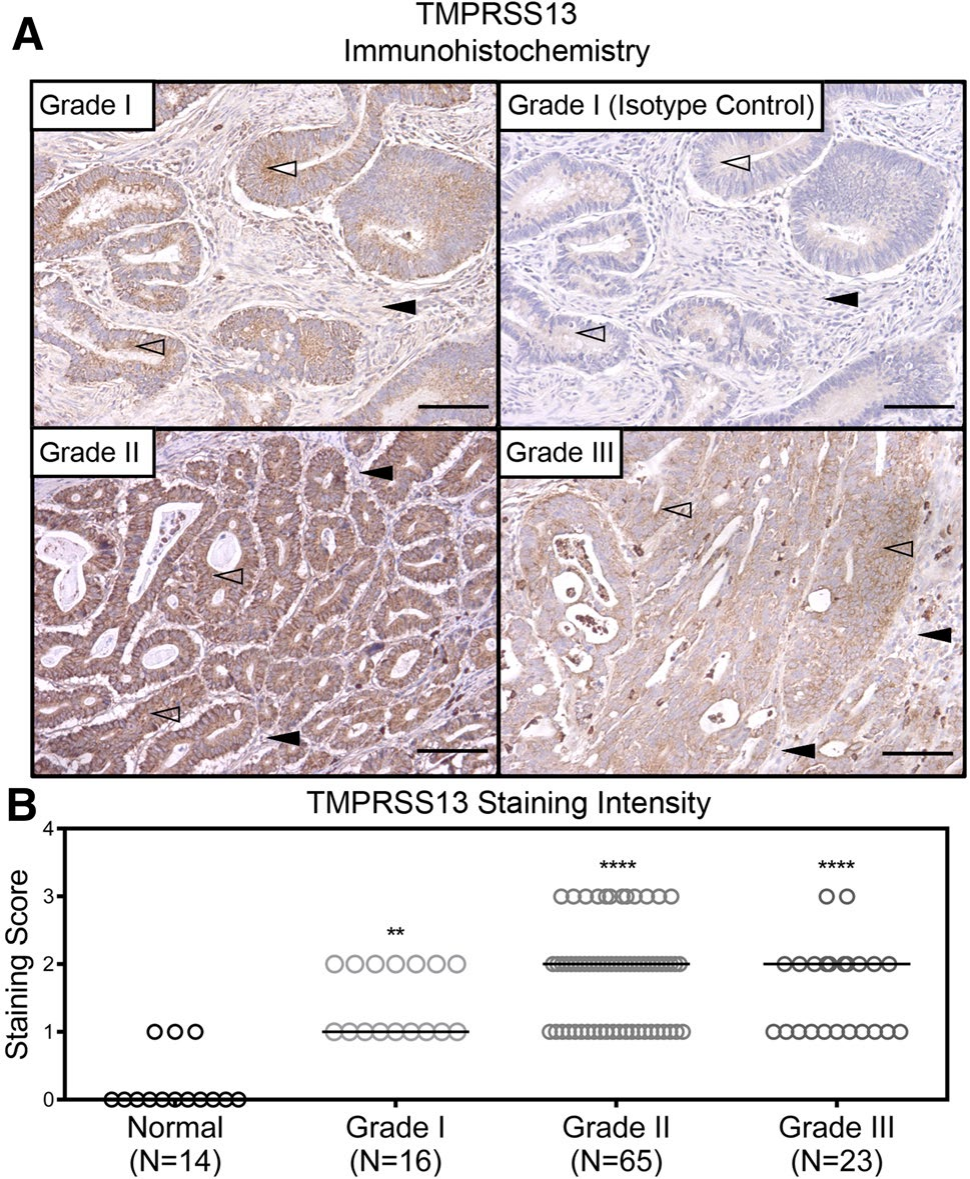
TMPRSS13 protein expression is upregulated in human colorectal cancer. (**A**) Representative samples from tissue array IHC analysis of TMPRSS13 protein expression in Grade I (upper left panel), Grade II (lower left panel), and Grade III (lower right panel) colorectal adenocarcinomas. Primary, rabbit anti-TMPRSS13 antibody was substituted with non-immune rabbit IgG in serial sections of all samples and no significant staining was observed (example in upper right panel). Open arrowheads indicate epithelial cells with weak or moderate TMPRSS13 staining in Grade I (upper left panel) compared to strong TMPRSS13 staining in epithelial cells of a Grade II (lower left panel), and weak or moderate TMPRSS13 staining in Grade III colon adenocarcinoma (lower right panel). The lamina propria is indicated with black arrowheads. Scale bars = 100 µm. (**B**) Staining intensities were determined as described in “Methods” and presented in a scatterplot categorized by cancer grade. Each circle represents one individual patient. Normal colon (N = 14), Grade I (N = 16), Grade II (N = 65), and Grade III (N = 23) colorectal adenocarcinoma. (**p < 0.01; ****p < 0.0001; determined by Dunn’s test posthoc, following Kruskal–Wallis ANOVA).

Evaluation of differential expression of TMPRSS13 in CRC was conducted using normal and cancerous tissue samples with grades ranging from I to III (Normal colon, N = 14; colorectal adenocarcinomas Grade I, N = 16; Grade II, N = 65; and Grade III, N = 23). The CRC tissue arrays were incubated with anti-TMPRSS13 rabbit antibody (Fig. 2A, representative samples of each grade shown) and non-immune rabbit IgG as a negative control (Fig. 2A, upper right panel). Staining intensity was scored on a scale from 0 to 3 (see “Methods”). The majority of normal colon samples (11/14) displayed no detectable TMPRSS13 expression while three samples showed low expression (Fig. 2B). In contrast, all CRC samples were scored positive for TMPRSS13 staining (Fig. 2B). Well-differentiated, low-grade carcinomas (Grade I) showed low to moderate cell surface staining (Fig. 2A, upper right panel), while the majority of moderately differentiated carcinomas (Grade II) displayed moderate to strong cell surface staining (Fig. 2A, lower left panel). In poorly differentiated carcinomas (grade III), TMPRSS13 expression mainly localized to the cell surface, with some areas displaying dispersed staining (Fig. 2A, lower right panel) and the majority of samples showing low to moderate staining. Statistical analyses showed a significant increase in staining intensity in all CRC grades (I–III) in comparison to normal tissue (Fig. 2B).

Together, these findings demonstrate differential TMPRSS13 protein expression in CRC, validating increased levels of transcripts as being accompanied by increased protein levels, and indicating a proteolytic imbalance in the colorectal tumor microenvironment.

### Loss of TMPRSS13 induces apoptosis in CRC cells

**Figure 3:**
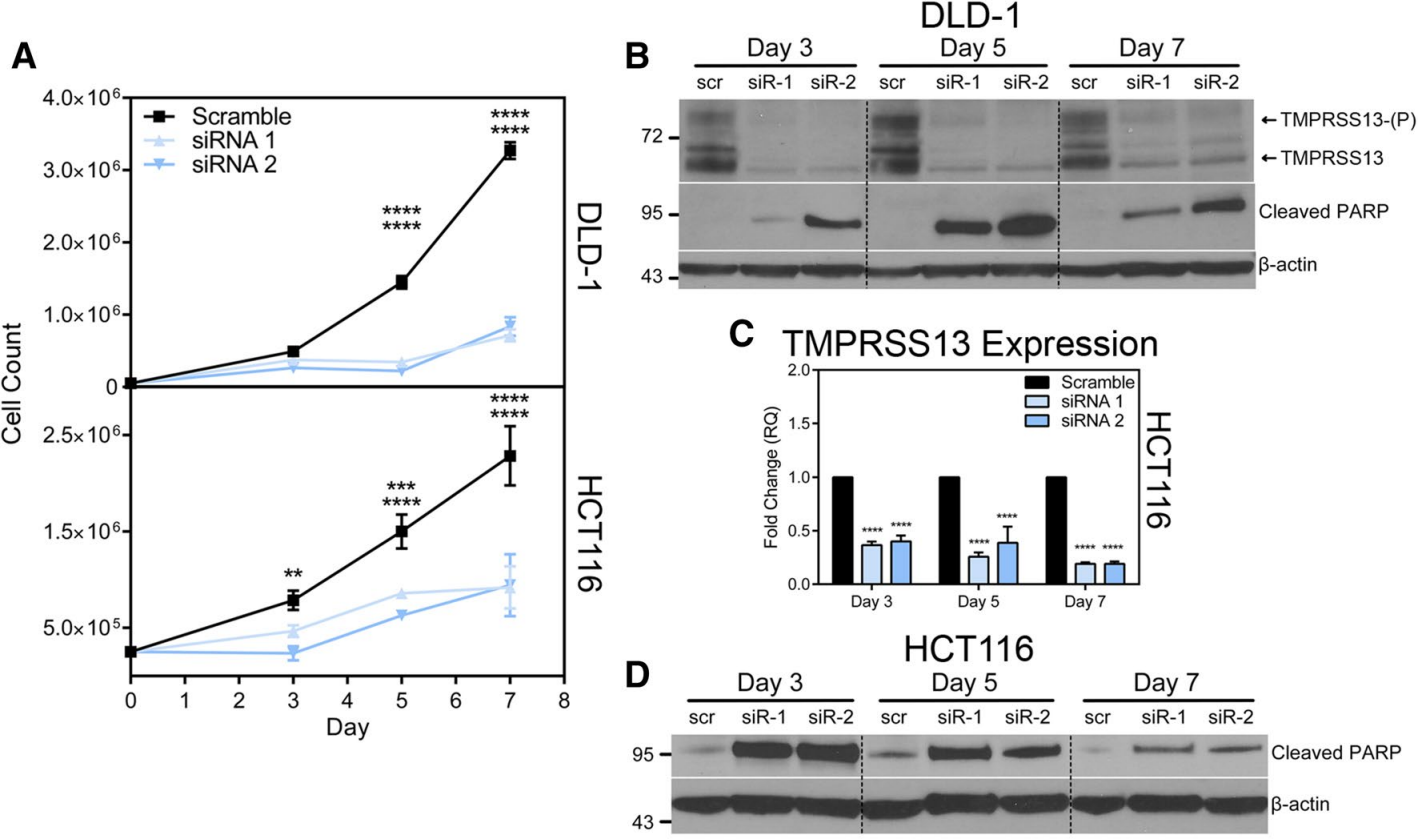
Silencing of TMPRSS13 decreases cell survival and leads to increased apoptosis in colorectal carcinoma cells. (**A**) TMPRSS13 was silenced using two non-overlapping synthetic RNA duplexes (siRNA 1 and siRNA 2) in the human colorectal carcinoma cell lines DLD-1 (top panel) and HCT116 (bottom panel) and cells were counted on day 3, day 5, and day 7 following siRNA treatment. A %GC-matched non-targeting RNA duplex was used as a negative control (Scramble). The number of viable cells counted was plotted for each time point. Error bars indicate SD (***p < 0.001; ****p < 0.0001, determined by Tukey’s posthoc test, following two-way ANOVA). (**B**) Verification of TMPRSS13 knockdown in DLD-1 cells was performed by western blot analysis. The high molecular weight band labeled TMPRSS13-(P) represents the phosphorylated form of TMPRSS13. The lower molecular weight bands (at or below the 72 kDa marker) labeled TMPRSS13, likely represent different glycosylation forms or isoforms of full-length TMPRSS13. Apoptosis was assessed by probing for cleaved PARP (middle panel) and anti-ß-actin was used as a control for equal loading (bottom panel). Dashed lines indicate cropped lanes. (**C**) Verification of TMPRSS13 knockdown in HCT116 cells was performed by qRT-PCR analysis using fold-change analysis normalized to HPRT1. Error bars indicate SEM (****p < 0.0001, determined using a one-way ANOVA with Tukey posthoc tests). (**D**) Apoptosis following TMPRSS13 knockdown was assessed by detection of cleaved PARP in HCT116 cells by western blot analysis (top panel). Anti-ß-actin was used as a control for equal loading (bottom panel). Dashed lines indicate cropped lanes.

Based on our observations that TMPRSS13 is upregulated in human CRC on the transcript and protein levels, we set out to determine the role of TMPRSS13 in pro-oncogenic cellular processes by using two different human CRC cell lines. The DLD-1 (high TMPRSS13 expression) and HCT116 (low TMPRSS13 expression) cell lines are both derived from colorectal adenocarcinomas. DLD-1 cells harbor mutations in the *KRAS, PIK3CA, APC,* and *TP53* genes. HCT116 cells harbor mutated *KRAS* and *PIK3CA,* and wildtype *APC* and *TP53* genes^29^. Both cell lines grow primary tumors upon orthotopic microinjection in nude mice with dissemination of cancer cells to local and distant sites^30^. To assess the effects of TMPRSS13 loss-of-function on cell survival, two non-overlapping siRNAs targeting TMPRSS13 were used and cells were counted at different time points after transfection. A significant decrease in the number of viable TMPRSS13-silenced cells was observed beginning three days post-siRNA transfection in HCT116 cells and five days post-siRNA transfection in DLD-1 cells compared to cells transfected with a scrambled %GC matched control siRNA (Fig. 3A). TMPRSS13-silencing was confirmed in DLD-1 cells by western blotting (Fig. 3B), whereas qRT-PCR analysis was used to verify silencing of TMPRSS13 in HCT116 (Fig. 3C) due to markedly lower baseline expression levels in this cell line, which led to unreliable detection of TMPRSS13 by western blotting (See Supplementary Fig. 2, empty vector lanes; other supportive data not shown). The multiple bands (~ 65–75 kDa) observed by western blot analysis in Fig. 3B may represent different isoforms of TMPRSS13, as five isoforms produced by alternative splicing have been reported^20^ and/or differential glycosylation of one or more of these isoforms. The size differences between MSPL, isoform 1, and isoform 4 are predicted to result in marginal migration differences (Supplementary Fig. 6 and Supplementary Table). We have previously reported that TMPRSS13 is subject to post-translational modification by glycosylation and phosphorylation^31^. The dominant TMPRSS13 form detected at ~ 70 kDa represents a glycosylated full-length form of TMPRSS13 and the species detected as a band of ~ 90 kDa represents a glycosylated, phosphorylated form of TMPRSS13 (TMPRSS13-(P))^31^. We previously identified these forms in multiple cancer cell lines, including DLD-1^31^.

**Figure 4:**
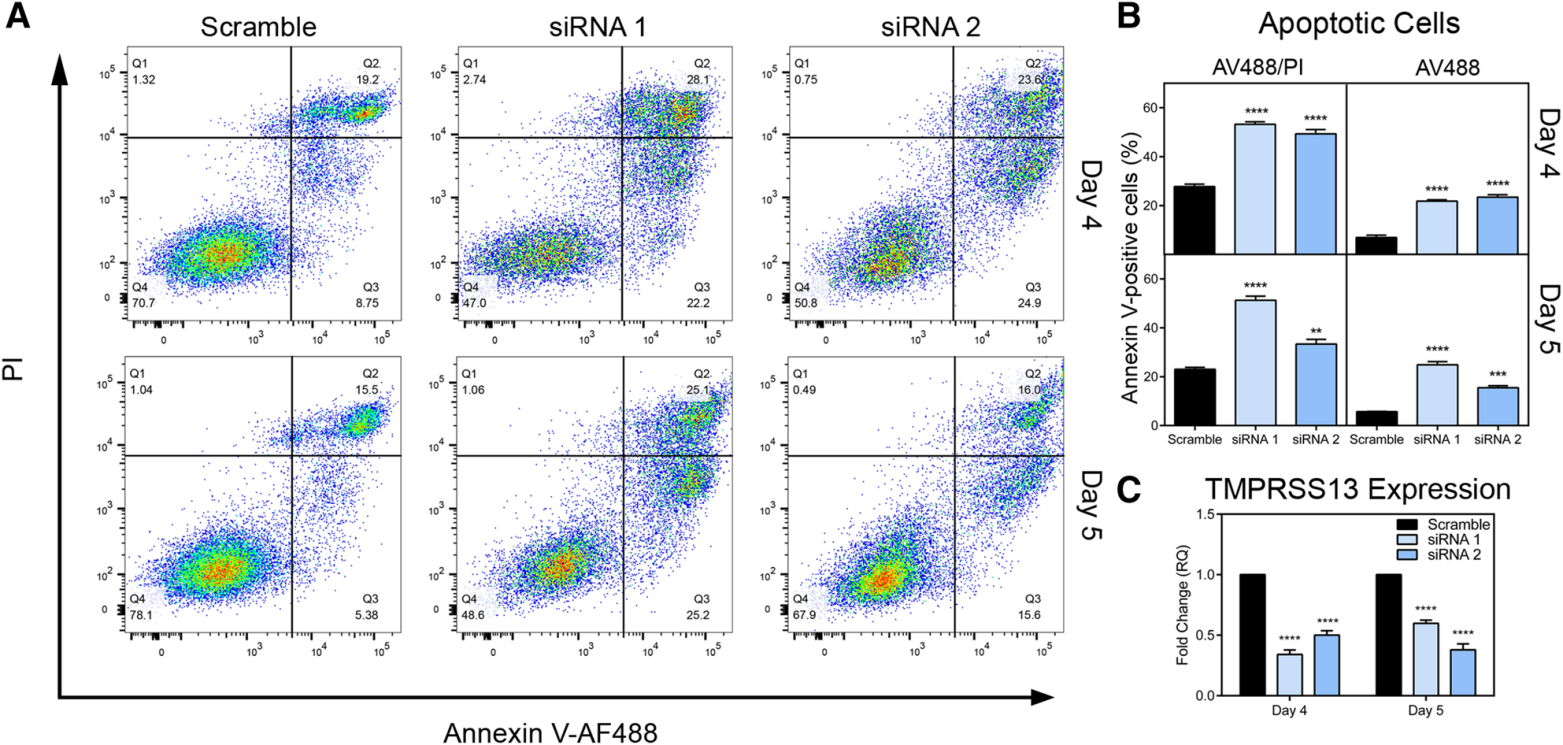
Flow cytometry analysis indicates increased apoptosis in colorectal carcinoma cells upon TMPRSS13 silencing. (**A**) Apoptosis in HCT116 cells was quantified by flow cytometry using an Annexin V-Alexa Fluor-488 conjugate (AV488) and propidium iodide (PI) at 4 days and 5 days post-siRNA treatment. Dot plots summarizing populations stained with AV488 and PI staining are shown for two non-overlapping synthetic RNA duplexes (siRNA 1 and siRNA 2). A %GC-matched non-targeting RNA duplex was used as a negative control (Scramble). Unstained cells are in Q4, with early-apoptotic AV488-positive stained cells being in Q3, late-apoptotic AV488/PI-positive stained cells captured in Q2, and necrotic PI-stained cells in Q1. (**B**) Analysis of AV488-positive populations, encompassing both early- and late-apoptotic cells (AV488+/PI + plus AV488+/PI-, left panels) and early apoptotic cells alone (AV488+/PI-, right panels), shows a significant increase in apoptotic cells among TMPRSS13 siRNA-treated cells. Error bars indicate SEM. (**p < 0.01; ***p < 0.001; ****p < 0.0001, compared to scramble control, determined by Tukey posthoc test, following one-way ANOVA). (**C**) qRT-PCR analysis of TMPRSS13 expression normalized to HPRT1 in HCT116 cells was used to confirm gene silencing. Error bars indicate SEM (****p < 0.0001).

To further characterize the cellular effects of TMPRSS13 knockdown, we assessed the level of cleaved Poly (ADP-ribose) polymerase (PARP) by western blotting as a marker for cells undergoing apoptosis in cell lysates. Robust increases in cleaved PARP levels were observed at all three time points (days 3, 5, and 7) upon TMPRSS13 silencing in both DLD-1 cells (Fig. 3B) and HCT116 cells (Fig. 3D). For a detailed comparative analysis of TMPRSS13-deficient cell populations versus TMPRSS13-sufficient cell populations, cells were stained with Annexin V-AlexaFluor™ 488 conjugate (AV488) in conjunction with the vital dye propidium iodide (PI) for analysis by flow cytometry to identify early- and late-stage apoptotic cells. The flow cytometric AV488/PI analysis data shows that a significantly higher proportion of TMPRSS13-silenced HCT116 cells underwent apoptosis compared to control cells (Fig. 4A, B left panels). The largest relative difference was observed in early apoptotic cells (AV488-positive/PI-negative) following TMPRSS13-silencing (Fig. 4B, right panels). The relative fractions of early apoptotic cells were: 22% for siRNA1 and 25% for siRNA2 versus 9% for control (day 4) and 25% for siRNA1 and 16% for siRNA2 versus 5% for control (day 5) (Fig. 4B, right panels). Silencing of TMPRSS13 in HCT116 was verified by qRT-PCR (Fig. 4C). DLD-1 cells were not amenable to the AV488/PI assay due to poor annexin-V staining, which is in line with previous reports^32^. Together, the decrease in viable cells, increased PARP-cleavage, and a higher relative apoptotic cell population revealed by flow cytometry analysis following TMPRSS13-silencing suggests an important role for TMPRSS13 in CRC cell survival and apoptosis.

### Increased TMPRSS13 expression promotes resistance to drug-induced apoptosis in CRC cells

**Figure 5:**
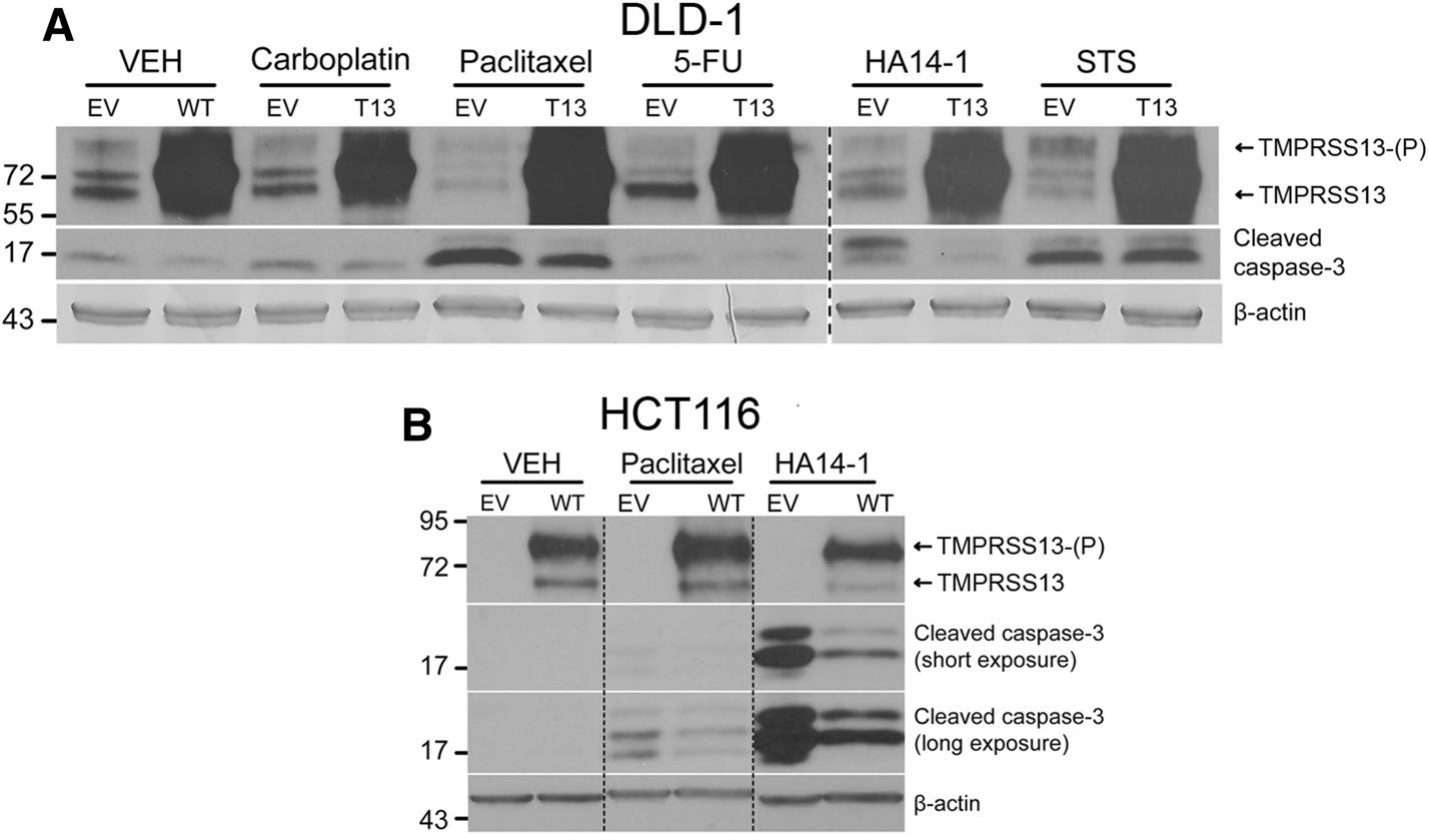
Overexpression of TMPRSS13 confers resistance to drug-induced apoptosis in colorectal cancer cells. (**A**) DLD-1 cells were transfected with a mammalian expression vector containing full-length, human TMPRSS13 (T13) cDNA (GenBank accession no. AAI14929.1). Full-length TMPRSS13 is shown, with and without phosphorylation (as indicated by (P)). As a control, cells were transfected with the same expression vector without the TMPRSS13 cDNA insert (EV). Twenty-four hours after transfection, cells were treated for 48 h with 50 µM carboplatin, 10 µM paclitaxel, or 100 µM 5-fluorouracil (5-FU); 10 µM HA14-1 for 1.5 h; or 1 µM staurosporine (STS) for 4 h. VEH = vehicle control cells. Cell lysates were collected 72 h post-transfection. TMPRSS13 overexpression was verified by western blotting (top panel). Apoptosis was examined by the detection of cleaved caspase-3 (middle panel) and anti-ß-actin was used as a control for equal loading (bottom panel). The break with the vertical heavy dashed line separates images from different blots. (**B**) HCT116 cells were transfected as described for DLD-1 cells to express TMPRSS13 and treated with 10 µM paclitaxel for 48 h, or 60 µM HA14-1 for 1 h. Cellular lysates were collected 72 h post-transfection and analyzed for TMPRSS13 expression and the presence of cleaved caspase-3 by western blot analysis. Anti-ß-actin was used as a control for equal loading (bottom panels). Light dashed vertical lines indicate cropping within one membrane.

Spurred by the finding that TMPRSS13 expression is elevated in CRC and that loss of expression results in an apoptotic response, we performed gain-of-function experiments to determine whether overexpression of the protease is sufficient for cells to acquire protective properties against apoptotic stimuli (Fig. 5). For this purpose, we utilized a panel of drugs including the chemotherapeutic drugs paclitaxel, carboplatin, and 5-FU; the broad-spectrum kinase inhibitor staurosporine; and the small-molecule Bcl-2 inhibitor HA14-1. These drugs cause cytotoxicity in a variety of cells through different mechanisms, but with apoptosis as the major outcome^33–37^. Full-length human TMPRSS13 (isoform 1; see “Methods”) was overexpressed in DLD-1 and HCT116 cells using transient plasmid transfection and confirmed by western blot (Fig. 5A,B, upper panels). The responses to drug treatment were assessed by western blot detection of caspase-3 cleavage (Fig. 5A,B, middle panels). Overexpression of TMPRSS13 led to a profound decrease in detected cleaved caspase-3 in CRC cells in response to HA14-1 in both cell lines. Thus, both DLD-1 cells, which have a relatively high level of endogenous TMPRSS13, and HCT116 cells with relatively low endogenous TMPRSS13 levels (Fig. 5A,B) were protected from HA14-1-induced apoptosis upon increased TMPRSS13 expression. Both cell lines also displayed TMPRSS13-mediated protection from apoptosis upon paclitaxel treatment. TMPRSS13 overexpression had no detectable effect on caspase-3 cleavage upon treatment with carboplatin, 5-FU, or staurosporine. The observed lack of response to 5-FU, which is widely used in advanced CRC, emphasizes a major clinical obstacle posed by acquired resistance to this and other chemotherapy drugs, which occurs in 90% of patients with metastatic cancer^33,38^.

### Loss of TMPRSS13 renders CRC cells more sensitive to drug-induced apoptosis

**Figure 6:**
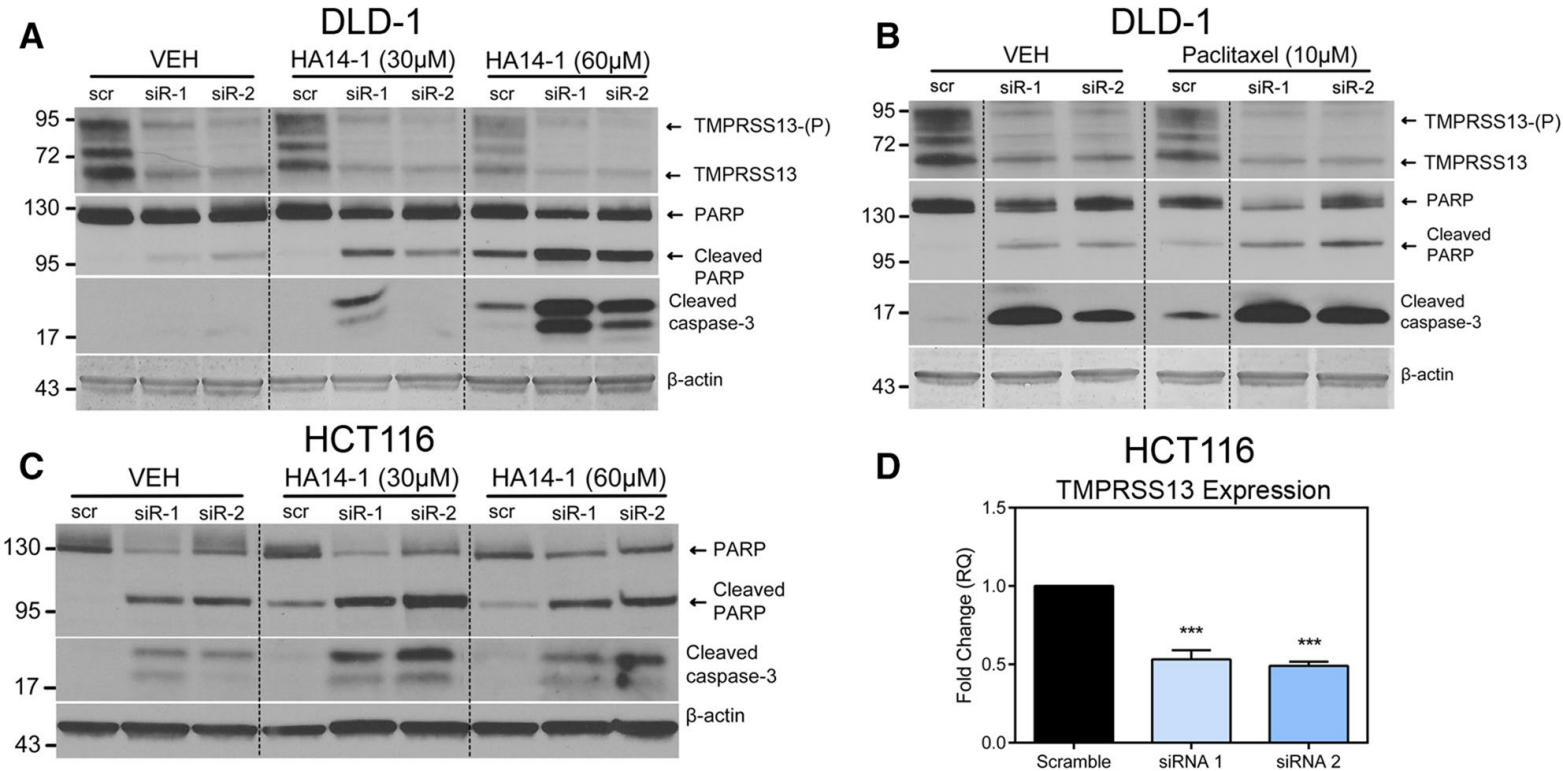
TMPRSS13 silencing sensitizes colorectal cancer cells to drug-induced apoptosis. Whole-cell lysates from DLD-1 (**A**, **B**) and HCT116 cells (**C**) were collected 96 h following siRNA treatment using two non-overlapping synthetic RNA duplexes (siRNA 1 and siRNA 2 = siR-1 and siR-2) targeting TMPRSS13. A %GC-matched RNA duplex was used as a negative control (scr). Before lysis, cells were subject to treatment with HA14-1 for 1 h (30 or 60 µM) or paclitaxel for 48 h (10 µM) or vehicle (VEH) as indicated. (**A**, **B**) Western blot analysis was used to verify TMPRSS13 knockdown in DLD-1 cells (top panels) and cleaved PARP and cleaved caspase-3 were used as markers of apoptosis in DLD-1 (middle panels). The high molecular weight band labeled TMPRSS13-(P) represents the phosphorylated form of TMPRSS13. The lower molecular weight bands (at or below the 72 kDa marker) labeled TMPRSS13, likely represent different glycosylation forms/isoforms of full-length TMPRSS13. Anti-ß-actin was used as a control for loading (bottom panels). Dashed lines indicate cropped membranes. (**C**) HCT116 cells treated as described above with HA14-1 or vehicle, respectively (**D**) qRT-PCR analysis of TMPRSS13 expression normalized to HPRT1 was used to verify gene silencing in HCT116 cells. Error bars indicate SEM (****p < 0.0001, determined using a one-way ANOVA with Tukey posthoc tests).

To further investigate the role of TMPRSS13 in drug-induced apoptosis, we investigated whether silencing of TMPRSS13 would increase sensitivity to this process, thereby exploring the potential of a novel combination treatment strategy using TMPRSS13 inhibitors together with chemotherapy to improve treatment efficacy (Fig. 6). HA14-1 and paclitaxel were selected based on the protective effect that TMPRSS13 overexpression had against these two apoptosis-inducing agents (Fig. 5).

HA14-1 or paclitaxel was added to TMPRSS13-silenced cells and levels of cleaved PARP and caspase-3 were detected by western blotting (Fig. 6A–C, middle panels). TMPRSS13 knockdown was confirmed by western blotting for DLD-1 (Fig. 6A,B, top panels) and by qRT-PCR for HCT116 cells (Fig. 6D). As expected, silencing of TMPRSS13 alone induced apoptosis in DLD-1 cells (Fig. 6A,B) and HCT116 cells (Fig. 6C). Importantly, HA14-1 treatment in combination with TMPRSS13 silencing further increased the level of apoptotic markers, indicating that the combination elicits a stronger apoptotic response than targeting either Bcl-2 (by HA14-1 treatment) or TMPRSS13 individually. The apoptotic response in DLD-1 cells to paclitaxel treatment was also enhanced in TMPRSS13-siRNA treated cells, although the potentiation by combination targeting compared to single targeting was less pronounced (Fig. 6B).

### Loss of TMPRSS13 reduces invasive potential of CRC cells

**Figure 7:**
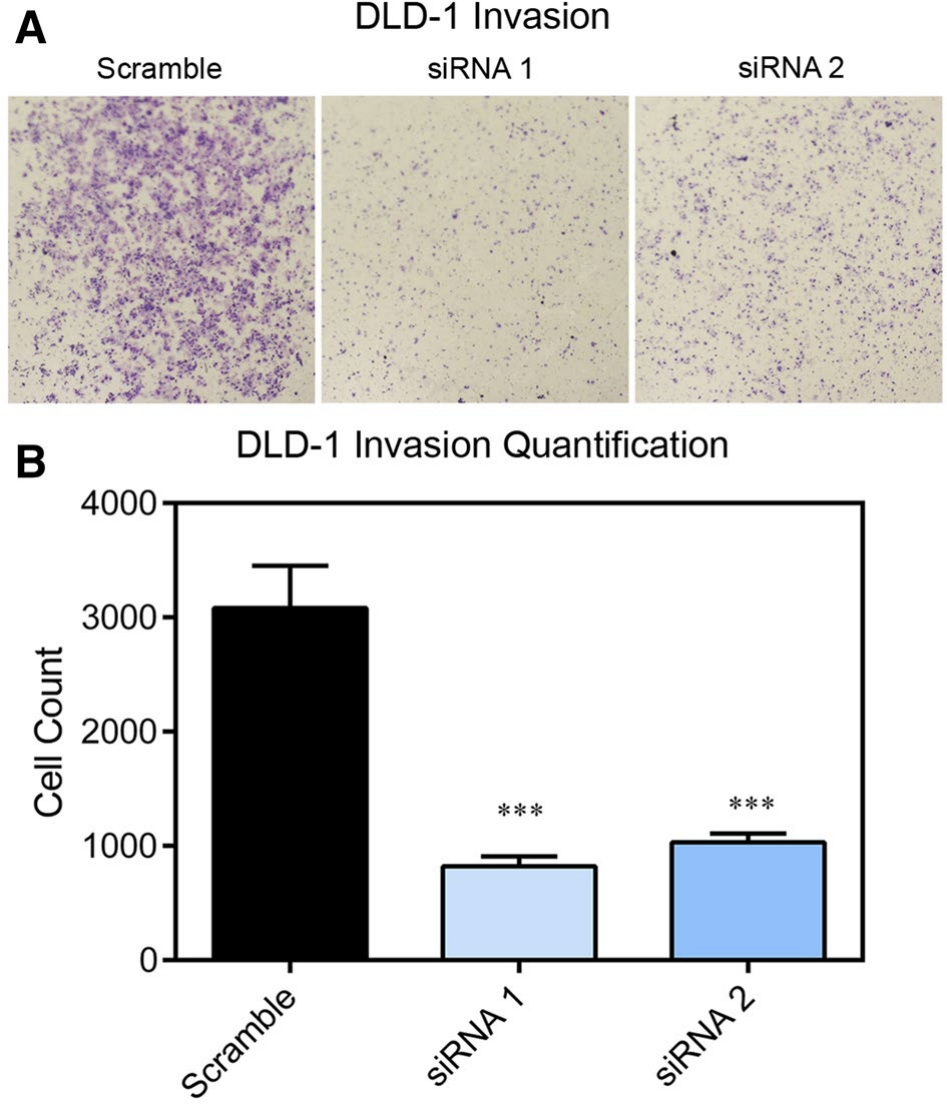
TMPRSS13 silencing reduces the invasive potential of colorectal cancer cells. (**A**) Invasion assays were performed in siRNA-treated DLD-1 cells using two non-overlapping synthetic RNA duplexes (siRNA 1 and siRNA 2) targeting TMPRSS13 and a %GC-matched RNA duplex as a negative control (Scramble). 48 h following siRNA treatment, DLD-1 cells were seeded in serum-free media onto transwell inserts coated with 1 mg/mL Cultrex® basement membrane gel, inserted into 24-well plates with serum-containing media. Cells were incubated for 16 h and invading cells were fixed and stained. Representative images of Cultrex®-coated transwell membranes containing invading cells are shown. (**B**) Invading cells were counted, and numbers analyzed by ANOVA, with Tukey’s posthoc test for multiple comparisons. Error bars indicate SEM (n = 3, ***p < 0.001).

For progression from localized to advanced/metastatic CRC to occur, cancer cells acquire properties consistent with a propensity to invade into surrounding tissues and distal organs. The role of TMPRSS13 for the invasive potential of CRC cells was assessed using a transwell assay in which cells were seeded on top of an extracellular matrix hydrogel in serum-free media and allowed to invade overnight towards full-serum media in the bottom chamber (Fig. 7). Upon silencing of TMPRSS13, a significant decrease in invasive potential was observed in DLD-1 cells (Fig. 7). Importantly, the experiment was carried out on day 2 after siRNA transfection when no difference in cell number is observed between control and TMPRSS13-silenced DLD-1 cells (Fig. 2A), minimizing interference from differences in cell proliferation/survival. These data suggest that TMPRSS13, in addition to anti-apoptotic functions, also promotes cellular invasion, two key pro-oncogenic functional properties involved in primary tumor growth and metastasis.

## Discussion

After seeking to identify uncharacterized TTSPs dysregulated in cancers, we discovered that the TMPRSS13 transcript is upregulated in CRC. Immunohistochemistry indicated increased TMPRSS13 protein levels in both well-differentiated and poorly differentiated cancers, suggesting that this protease is a potential promoter of CRC progression. Through further characterization of TMPRSS13 in cell culture models, we found that TMPRSS13 expression promoted cellular survival and invasive potential in CRC cell lines. Flow cytometric analysis revealed a significant increase in Annexin V-positive/PI-negative TMPRSS13-silenced cells, indicative of early apoptotic stages. In colon epithelia, apoptosis is a tightly regulated process crucial to the maintenance of epithelial integrity^39^. Suppression of apoptosis in cancer is critical for tumor progression and various pathways are altered to facilitate escape from cell death^40^. Our findings suggest that TMPRSS13 is a key player in promoting resistance to apoptosis in CRC cells. These properties of TMPRSS13 have not previously been described in any cancer type. A recent study demonstrated that expression of another member of the Hepsin/TMPRSS subfamily of TTSPs, TMPRSS4, correlates with colorectal cancer pathological stage^41^. Furthermore, RNA-mediated silencing experiments in HCT116 cells revealed that TMPRSS4 is involved in the regulation of cell proliferation, apoptosis, and invasion^41^. It is plausible that the two closely related proteases both promote CRC progression.

Identifying novel key players in CRC progression and their functions on the cellular level is a critical step towards the development of potential new targeted therapies. On the mechanistic level, a major challenge in current protease research is determining their mechanism of action, including the identification of physiologically relevant substrates. Many proteases, including TTSPs, are capable of cleaving multiple substrates in vitro^14–18^. Approaches to identify critical substrates for TTSPs and other extracellular/pericellular proteases include biased experiments where the effect of genetic ablation or inhibition of the protease in question on potential substrate cleavage is assessed. Alternatively, mass spectrometry-based approaches for determining the proteolytic events in complex biological samples (degradomics) have been used to discover substrates and potential cleavage sites^42^. However, understanding the physiological relevance of these results remains a significant challenge, and substrate candidates identified in these experiments must still be rigorously validated in cell-based assays or animal models^42^. Because the biochemical and cell biological features of TMPRSS13 are still relatively uncharacterized and have not been studied in the context of cancer, the only two candidate mammalian substrates for TMPRSS13 reported to date are pro-HGF and ENaC^25,26^. HGF is biosynthesized as the single-chain zymogen-like inactive precursor, pro-HGF, and it has been shown that TMPRSS13 can proteolytically process pro-HGF into its two-chain form in a cell-free system^25^. HGF is a pleiotropic growth factor and a key mediator of cell migration/invasion, proliferation, and survival/apoptosis in cancer cells via activation of its receptor, c-Met^43^. It has previously been reported that matriptase is the main activator of pro-oncogenic HGF/c-Met in in vivo models of breast and squamous cell carcinoma^44,45^. Furthermore, pertaining specifically to CRC, inhibition or silencing of matriptase in DLD1 cells efficiently impaired the conversion of pro-HGF into active HGF at the cell surface and inhibited cell scattering upon pro-HGF stimulation^46^. Matriptase is highly expressed in CRC patient samples and in CRC cell lines, including those used for this study^46,47^, which makes it plausible that matriptase is the major activator of pro-HGF in this cancer type. However, it cannot be ruled out that other proteases, including TMPRSS13, contribute to pro-HGF activation in CRC under certain physiological or pathological conditions. The other candidate substrate, human ENaC, was shown to be activated by TMPRSS13 in a *Xenopus laevis* cellular assay^26^ and activation of ENaC in cancer cells has been implicated in regulation of cellular survival/apoptosis (see further discussion below)^48^.

Despite advances in systemic therapies, the five-year survival rate for metastatic CRC remains below 15%^49^, making novel approaches to combat late-stage disease necessary, including the development of novel targeted therapies. This prompted us to test whether TMPRSS13 contributes to a drug-resistant phenotype in CRC cells. Indeed, upon overexpression of TMPRSS13, CRC cells exhibited resistance to treatment with the apoptosis-inducing drugs HA14-1 and paclitaxel. Conversely, TMPRSS13-silenced cells exhibited increased sensitivity to cell death induced by HA14-1 and, to a lesser extent, paclitaxel. Taxanes, including paclitaxel, have failed to demonstrate significant clinical benefit in phase II trials in CRC and are not used as standard-of-care^50,51^. In tissue culture experiments using SW480 and DLD-1 cells, paclitaxel-induced apoptosis can be enhanced by simultaneous inhibition of the mitogen-activated protein kinase (MAPK) pathway in CRC^52^. Thus, the treatment of SW480 and DLD-1 cells with paclitaxel resulted in increased activation of the MAPK pathway. In both cell lines, MAPK attenuation by PD98059, a MEK inhibitor, led to an enhancement of paclitaxel-induced apoptosis^52^. Synergistic inhibition of colon cancer cell growth with paclitaxel and the PI3K/mTOR dual inhibitor BEZ235 through apoptosis in HCT116 and HT-29 colon cancer cells has also been reported^53^. Further studies are needed to determine whether paclitaxel in combination with other drugs, including TMPRSS13 inhibitors, could be a viable therapeutic strategy in CRC.

In our studies, we observed the most profound apoptotic effect when combining TMPRSS13 silencing with HA14-1 treatment, which provided a rational starting point to explore potential mechanisms. Based on the selective action of HA14-1 on Bcl-2^35,54^, we investigated whether Bcl-2 levels were affected upon TMPRSS13 silencing. While decreases in Bcl-2 expression upon TMPRSS13 silencing were frequently observed, this phenomenon was not consistently detected under all cell culture conditions; therefore, we could not definitively confirm a functional relationship between TMPRSS13 and Bcl-2 (data not shown). Crosstalk between TMPRSS13 and Bcl-2 associated pathways may contribute to the increased apoptotic effect we observed; the implications of such a connection are significant, as Bcl-2 is often upregulated in cancer, including CRC^55^. Bcl-2 promotes Akt signaling independent of mitochondrial involvement^56^; however, as a cell-surface protease, a point of entry for TMPRSS13 into this pathway remains elusive. In this context, it should be mentioned that TMPRSS13 differs from other TTSPs by having a large intracellular domain with tandem repeat phosphorylation motifs of various protein kinases^57^, and it has been demonstrated that TMPRSS13 is phosphorylated in cancer cells, including DLD1 cells^31^. The roles of TMPRSS13 phosphorylation in pro-oncogenic signaling, including regulation of apoptosis, are currently under investigation.

It is noteworthy that inhibitors targeting the Bcl-2 protein have gained attention in recent years. In 2016 the FDA approved venetoclax, a selective small-molecule inhibitor of Bcl-2, for treatment of chronic lymphocytic leukemia (CLL). Particularly in hematological malignancies, multiple clinical trials are assessing the possible use of venetoclax, alone or in combination with other chemotherapies^58^. It remains to be seen whether the positive results observed in diseases like CLL using anti-apoptotic inhibition will be mirrored in solid tumors, including CRC, that are commonly more difficult to treat in the clinic. Promising results have recently been reported on venetoclax when combined with tamoxifen in ER/Bcl-2-positive metastatic breast cancer with an overall response rate of 61% and a 72% clinical benefit rate^59^, which underscores the importance of testing new combination therapies.

Ion channels, including calcium and sodium channels, play a critical role in cancer by regulating cell survival and apoptosis^60,61^. HA14-1 and paclitaxel can both facilitate increased intracellular calcium levels, albeit by different mechanisms, which trigger mitochondria-mediated and ER stress-associated apoptotic cascades. In our present study, these two drugs enhanced the cellular death observed in combination with TMPRSS13 knockdown^62–67^. With both HA14-1 and paclitaxel increasing intracellular calcium levels, ion imbalances may potentiate the cellular death associated with TMPRSS13 silencing. One potential mechanism by which TMPRSS13 might affect intracellular ion levels is by proteolytic activation of ENaC. It was previously reported that increased ENaC-mediated sodium currents were measured in *Xenopus* oocytes injected with human ENaC and TMPRSS13 mRNA^26^. In cancer, it has been proposed that ENaC-mediated Na^+^ influx promotes survival, proliferation, and invasion of cancer cells, whereas blockade of Na^+^ influx via ENaC induces cell cycle arrest and apoptosis^48,61^. It can be speculated that TMPRSS13-mediated ENaC activation in CRC (ENaC is expressed in colon epithelial cells and datasets in Oncomine™ report high expression of ENaC in both DLD1 and HCT116 cells^68,69^) could contribute to survival and invasion, whereas silencing of the protease may decrease ENaC activation, leading to increased apoptosis and enhanced sensitivity to HA14-1 and paclitaxel.

Our studies indicate a role for TMPRSS13 in conferring cells with invasive potential and protecting cells from apoptosis, suggesting that TMPRSS13 is a potential therapeutic target in CRC. One factor to consider is the potential side effects of TMPRSS13 inhibition in vivo. TMPRSS13 is an attractive target because of its low baseline expression levels in the healthy colon, potentially limiting the adverse effects of TMPRSS13 inhibition. Furthermore, previous studies in mouse genetic loss-of-function models demonstrate that TMPRSS13 deficiency has no discernible consequences for adult mice^19^. Still, since the function of TMPRSS13 under challenged conditions remains uncharacterized, the impact of TMPRSS13 inhibition on the function and homeostasis of the colon and other tissues requires additional examination.

In summary, this study represents a comprehensive characterization of TMPRSS13 in CRC, highlighting cancer-associated dysregulation of this TTSP and underscoring it as a critical component of CRC cell survival and protection from drug-induced apoptosis. These findings lay the foundation for continuing studies to further decipher the molecular mechanisms by which TMPRSS13 exerts its pro-oncogenic functions, to promote the development of TMPRSS13 inhibitors, and to study their potential as targeted therapeutic drugs in CRC.

## Materials and methods

### In silico analysis

The Oncomine™ online platform (https://www.oncomine.org) was used to perform a meta-analysis of TMPRSS13 expression, comparing colorectal cancer samples to normal colon across multiple transcriptome-wide studies. Relevant datasets were identified utilizing the differential analysis; cancer vs. normal analysis; and TMPRSS13 gene filters. The results shown were based upon data generated by the TCGA Research Network: https://www.cancer.gov/tcga.

### Tissue samples, immunohistochemistry, and evaluation of staining

Colorectal (CO1921) tissue arrays containing both cancer and normal samples were obtained from US Biomax, Inc. Analysis of TMPRSS13 protein in normal human tissue for antibody validation was performed using human tissue arrays UNC241 and OR301 (US Biomax Inc.). Paraffin-embedded arrays were cleared in xylene (Fisher Scientific) and rehydrated in a graded series of ethanol solutions. For antigen retrieval, tissue arrays were boiled in reduced pH citrate antigen retrieval buffer (Vector Laboratories) for 10 min. Endogenous peroxidase activity was quenched by incubating tissue arrays in 3% H_2_O_2_ for 15 min. Arrays were blocked with 2.5% bovine serum albumin (Sigma Aldrich) in PBS for 1 h at room temperature and incubated overnight in anti-TMPRSS13 antibody (1:150, PA5-30935, Thermo Fisher Scientific) at 4 °C in a humidified chamber. All washing steps were performed with PBS. Non-immune rabbit IgG (Neomarkers) was used as a negative control. Visualization of bound primary antibody was performed using a biotinylated anti-rabbit secondary antibody and conjugated horseradish peroxidase H contained in the VECTASTAIN ABC kit (Vector Laboratories). Enzymatic visualization was carried out with 3,3-diaminobenzidine (DAB) substrate (Sigma-Aldrich) and arrays were subsequently counterstained with hematoxylin. Stained slides were washed and dehydrated in a series of graded ethanol solutions followed by xylene and mounted with glass coverslips using Permount (Thermo Fisher Scientific). Microscopic images were acquired on a Zeiss Axio Scope. A1 using digital imaging.

To evaluate staining intensity in colon tissue arrays, samples were assessed microscopically. Epithelial staining of TMPRSS13 was rated based on intensity in 20 × microscopic fields on a scale of 0 to 3, where: 0 = no epithelial staining; 1 = majority weakly stained epithelial cells OR few moderately stained epithelial cells among a majority of non-stained cells; 2 = majority moderately stained epithelial cells OR few strongly stained among a majority of weakly or non-stained cells; 3 = majority strongly stained epithelial cells.

### Cell lines and culture conditions

HCT116 cells (ATCC) were cultured in minimal essential media (MEM) (Gibco/Thermo Fisher Scientific) supplemented with 10% fetal bovine serum (FBS) (Atlanta Biologicals), 10 units/mL penicillin and 10 µg/mL streptomycin (Gibco/Thermo Fisher Scientific), and 1 × non-essential amino acids (Gibco/Thermo Fisher Scientific). DLD-1 cells (ATCC) were cultured with RPMI-1640 media supplemented with 10% FBS and 10 units/mL Penicillin and 10 µg/mL Streptomycin (Gibco/Thermo Fisher Scientific). Cells were maintained in a humidified incubator at 37 °C with an atmosphere of 5% CO_2_.

### RNAi-mediated gene silencing

For TMPRSS13 knockdown in DLD-1 and HCT116 colon cancer cells, two independent Stealth RNAi™ siRNA duplexes (Invitrogen/Thermo Fisher Scientific) targeting TMPRSS13 (siRNA 1 = HSS130531, siRNA 2 = HSS130532) were used. A matched %GC negative scramble control (12,935,300) was included in all experiments. Reverse transfections, in which siRNA-lipid complexes were added to wells before seeding, were performed using Lipofectamine RNAiMAX (Invitrogen/Thermo Fisher Scientific) following manufacturer instructions. Cells were transfected in 6-well plates for all experiments except for cell-counting experiments and flow cytometric analyses, in which 12-well plates were used and reagent levels adjusted according to transfection reagent manufacturer guidelines. Media was replaced every 48 h during siRNA treatment for all experiments. Cellular lysates were collected 3, 5, and 7 days post-transfection for proliferation experiments, 4 days post-transfection for drug treatment experiments, and 4 to 5 days post-transfection for flow cytometry experiments.

### Cell counting

DLD-1 and HCT116 cells were reverse transfected with siRNAs targeting TMPRSS13 and seeded onto 12-well plates at 50,000 cells/well for DLD-1 and 250,000 cells/well for HCT116. Cells were trypsinized 3, 5, and 7 days post-transfection, pelleted by centrifugation at 200×*g* for 5 min, and resuspended in media. Samples were mixed 1:1 with 0.4% trypan blue stain (Gibco/Thermo Fisher Scientific) to distinguish viable and dead cells. Counting was performed using a hemocytometer.

### Western blot analyses

Cultured DLD-1 or HCT116 cells were washed 3 times with ice-cold PBS and lysed in- well using ice-cold RIPA buffer (150 mM NaCl; 50 mM Tris/HCl, pH 7.4; 0.1% SDS; 1% NP-40) with protease inhibitor cocktail (Sigma Aldrich); and phosphatase inhibitor cocktail (Sigma Aldrich) and cleared by centrifugation at 12,000×*g* at 4 °C. Quantification of cell lysate protein concentrations was performed using the Pierce BCA Protein Assay Kit (Thermo Fisher Scientific). Lysates were prepared with SDS lysis buffer containing a reducing agent (50 mM Tris–HCl, pH 6.8, 0.25% bromophenol blue, 5% glycerol, 1.5% SDS, and 100 mM dithiothreitol) and boiled for 5 min before loading onto 4–15% Mini-Protean® or Criterion® TGX gels (Bio-Rad) for SDS-PAGE, followed by blotting onto 0.2 µm Immun-Blot® polyvinylidene difluoride membranes (Bio-Rad). Membranes were blocked with 5% (w/v) dry milk powder in TBS-T (Tris-buffered saline, 0.1% Tween-20) for 1 h at room temperature and subsequently incubated overnight at 4 °C in primary antibodies diluted in 5% dry milk powder/TBS-T. Primary antibodies used for western blotting included rabbit anti-TMPRSS13 (1:2000, ab59862, Abcam), rabbit anti-cleaved caspase-3 (1:500, 9661, Cell Signaling Technology), rabbit anti-PARP (1:1000, 9532, Cell Signaling Technology), rabbit anti-cleaved PARP (1:1000, 5625, Cell Signaling Technology), and mouse anti-β-actin (1:10,000, NB600-501, Novus Biologicals). Goat anti-rabbit and goat anti-mouse (Millipore) HRP-conjugated antibodies were used as secondary antibodies. Detection of antibodies was performed using ECL Western Blotting substrate or Super-Signal West Femto Chemiluminescent Substrate (Pierce, Thermo Fisher Scientific).

### Transient transfections with TMPRSS13 expression vector

Transient expression of TMPRSS13 in DLD-1 and HCT116 cells was performed in 6-well plates through reverse transfection with 500 ng of plasmid using Lipofectamine LTX (Invitrogen) according to manufacturer instructions. The pcDNA3.1 (Invitrogen/Thermo Fisher Scientific) plasmid vector lacking the coding sequence was used as an empty vector control, as well as pcDNA3.1-TMPRSS13 (human full-length)^31^, a plasmid containing the coding sequence for TMPRSS13 (GenBank accession no. AAI14929.1; see Supplementary Table for isoform comparison). This clone is representative of TMPRSS13 isoform 1 with a Q83-A87 (QASPA) deletion. The Catalogue of Somatic Mutations in Cancer (COSMIC) reports that in screens targeting TMPRSS13, nearly 13% of cancer samples are positive for a Q83-Q87 deletion of TMPRSS13. Notably, colorectal cancer makes up 41% of cases positive for this deletion^70^. Media was replaced 24 h following transfection and cells were subjected to drug treatment as described below. Cellular lysates were collected 3 days post-transfection in drug treatment experiments.

### Drugs and drug treatments

For drug treatment experiments, stock solutions of paclitaxel (Sigma Aldrich), 5-FU (Sigma Aldrich), HA14-1 (Cayman Chemical), and staurosporine (STS) (Cell Signaling Technology) were diluted in DMSO, whereas water was used to dilute a stock solution of carboplatin (Sigma-Aldrich). For the treatment of cells overexpressing TMPRSS13, DLD-1 cells were subjected to 48-h treatments with paclitaxel (10 µM), carboplatin (50 µM), or 5-FU (100 µM) starting 24 h after transient transfection with plasmid vectors. Twenty-four hours after transient transfection with an expression vector, DLD-1 and HCT116 cells were subjected to treatment with HA14-1 or STS. The duration of HA14-1 treatment for DLD-1 cells was 1.5 h at a concentration of 10 µM and a duration of 4 h for STS treatment at a concentration of 1 µM. In HCT116 cells, HA14-1 treatment lasted 1 h at 60 µM. All drug treatment conditions were concluded by lysate collection. For drug treatment of TMPRSS13-silenced cells, siRNA-treated DLD-1 cells were subjected to a 48-h treatment of paclitaxel at a final concentration of 10 µM (48 h post-transfection) and lysates were collected four days post-transfection. Four days post-transfection, siRNA-treated DLD-1 and HCT116 cells were subjected to a 1-h treatment of HA14-1 at final concentrations of 30 and 60 µM.

### Quantitative real-time polymerase chain reaction (qRT-PCR)

Total RNA was isolated from cultured cells using the RNeasy Plus Kit (Qiagen) according to manufacturer instructions. Reverse transcription of RNA isolates was performed using the High Capacity cDNA Reverse Transcription Kit (Applied Biosystems). The qPCR reactions were performed with probes for TMPRSS13 (Hs00361060_m1, TaqMan®, Applied Biosystems) and expression levels were analyzed using the -2^ΔΔCt^ method and normalized to HPRT1 or GAPDH (Hs02800695_m1 and Hs02758991_g1, TaqMan®, Applied Biosystems).

### Analysis of apoptosis by Annexin V/propidium staining and flow cytometry

Flow cytometry analysis was performed in the Wayne State University Flow Cytometry Core. Labeling of live HCT116 cells was performed using the Alexa Fluor® 488 Annexin V/Dead Cell Apoptosis Kit (Molecular Probes) according to manufacturer instructions. Specifically, siRNA-treated HCT116 cells were cultured in 12-well plates, with media replaced every 48 h. At four- and five-days post-transfection, cells were trypsinized with 0.25% Trypsin–EDTA (Gibco, Thermo Fisher Scientific), centrifuged at 200×*g* for 5 min, and cell pellets washed twice with ice-cold PBS. Following washes, 100 ul of cellular suspension from each biological sample was combined with 400 µL of 5 × annexin binding buffer and gently mixed. Cells were analyzed within 30 min of staining with an LSR II (Becton Dickinson) cytometer. Cytometric data were plotted and analyzed with FlowJo software.

### Invasion assay

Two days following siRNA-mediated TMPRSS13 silencing, DLD-1 cells were serum-starved for 5 h, and then seeded in serum-free media onto 1 mg/mL Cultrex®-coated (Trevigen, Gaithersburg, MD) permeable support inserts (8.0 µm pore size, Falcon). Inserts were placed in 24-well plates with serum-containing media as a chemoattractant and cells were cultured on inserts for 24 h, after which invading cells were fixed using Z-fix (Anatech, Battle Creek, MI) and stained using Diff-Quik (Siemens, Deerfield, IL). Images of inserts were acquired using an EZ4D Stereo Zoom microscope with an integrated digital camera (Leica Microsystems, Buffalo Grove, IL), and invading cells quantified from images using ImageJ software.

### Statistical analyses

All statistical analyses were performed using GraphPad Prism software. For immunohistochemical staining, differences in staining scores between cancer grade groups were analyzed using the non-parametric Kruskal–Wallis ANOVA test with posthoc comparisons performed with Dunn’s test. For proliferation experiments, differences between siRNA treatment groups were analyzed using the two-way ANOVA test, with siRNA treatment and time point as independent factors. Posthoc comparisons were performed between siRNA treatments for each time point using Tukey’s multiple comparisons test. One-way ANOVA tests with Tukey’s multiple comparisons posthoc tests were used for comparison of siRNA treatment groups in flow cytometry, invasion, and qRT-PCR experiments.

## Supplementary information

Supplementary Information.
