# Supplementary material for: TMPRSS13 promotes cell survival, invasion, and resistance to drug-induced apoptosis in colorectal cancer
Source: Sci Rep. 2020 Aug 17;10:13896. doi: 10.1038/s41598-020-70636-4 (PMC7431588; doi:10.1038/s41598-020-70636-4)
Supplement: Supplementary file 1 — Supplementary Information. [file 41598_2020_70636_MOESM1_ESM.pdf]

TMPRSS13 promotes cell survival, invasion, and resistance to drug-induced apoptosis in colorectal cancer

Fausto A. Varela, Victoria L. Foust, Thomas E. Hyland, Kimberley E. Sala-Hamrick, Jacob Mackinder, Carly E. Martin, Andrew S. Murray, Sokol V. Todi, Karin List

Supplementary Information

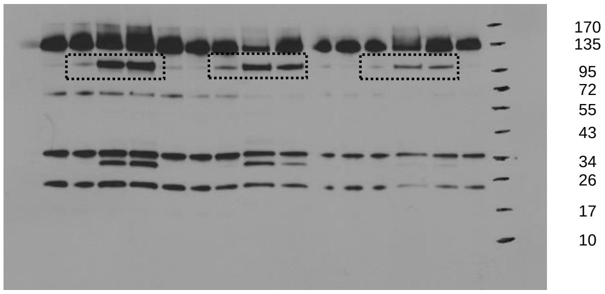

Anti-PARP

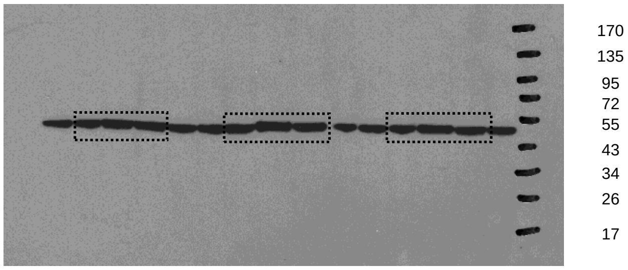

Anti-β-actin

Supplemental Figure 1: Non-cropped western blot exposures from experiments using HCT116 cells. X-ray film exposures of western blots used in Figure 3D. Dash-outline box indicates area used for figure.

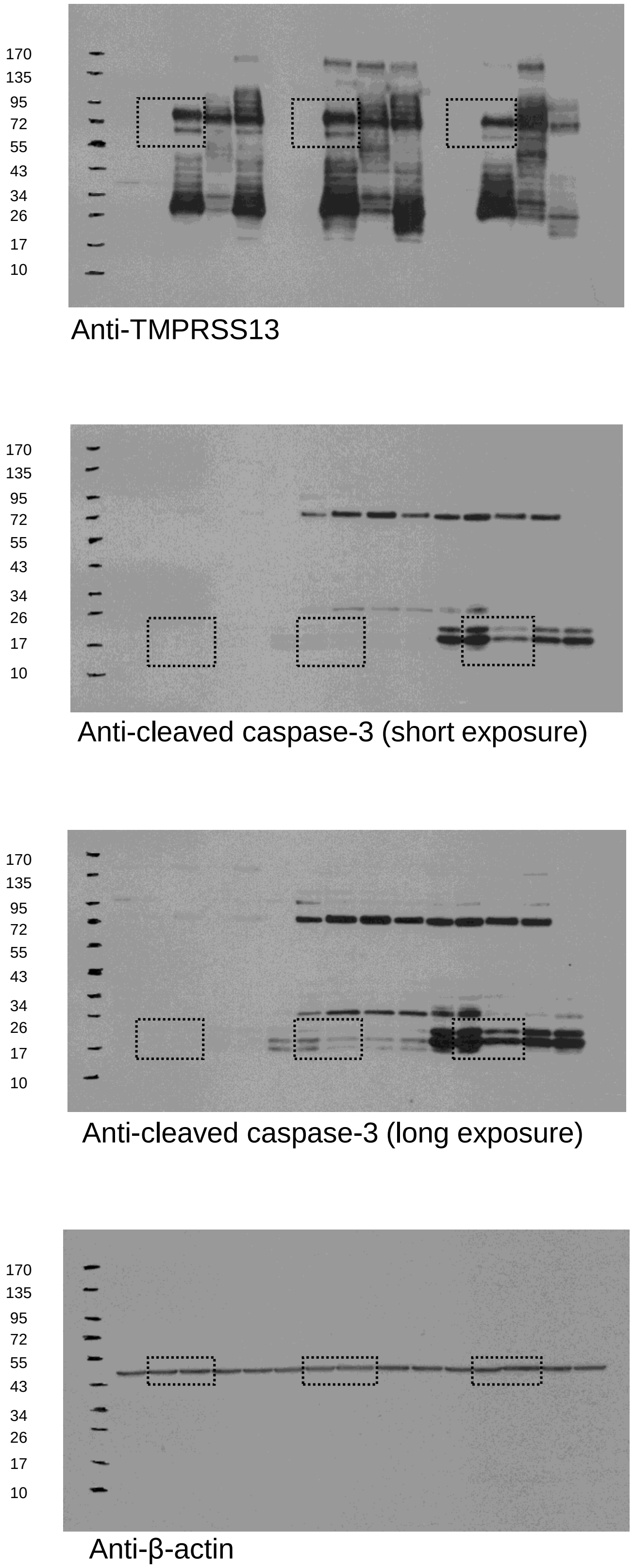

Supplemental Figure 2: Non-cropped western blot exposures from experiments using HCT116 cells. X-ray film exposures of western blots used in Figure 5B. Dash-outline box indicates area used for figure.

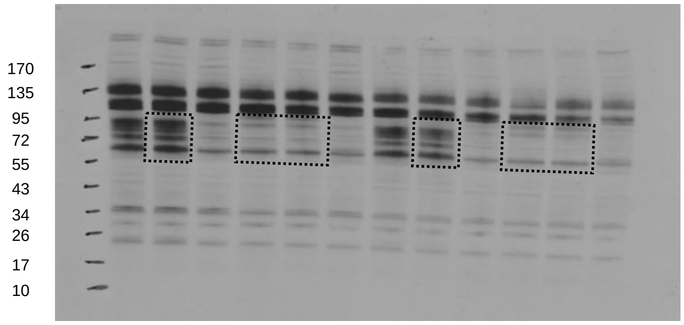

Anti-TMPRSS13

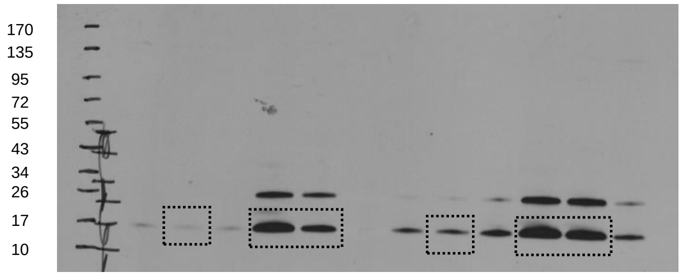

Anti-cleaved caspase-3

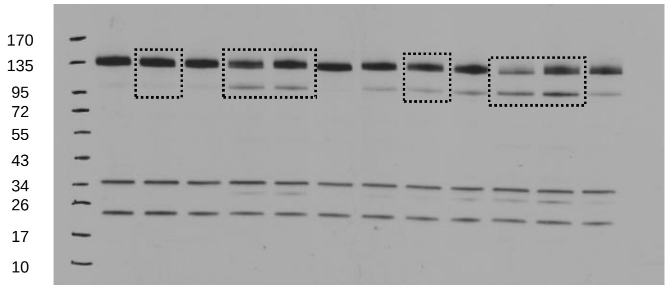

Anti-PARP

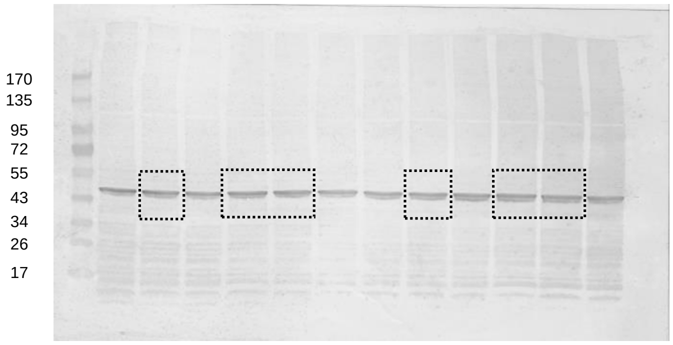

Anti-β-actin

Supplemental Figure 3: Non-cropped western blots from experiments using DLD-1 cells. X-ray film exposures of western blots used in Figure 6B. Bottom panel (Anti-β-actin) is the uncropped blot membrane. Dash-outline box indicates area used for figure.

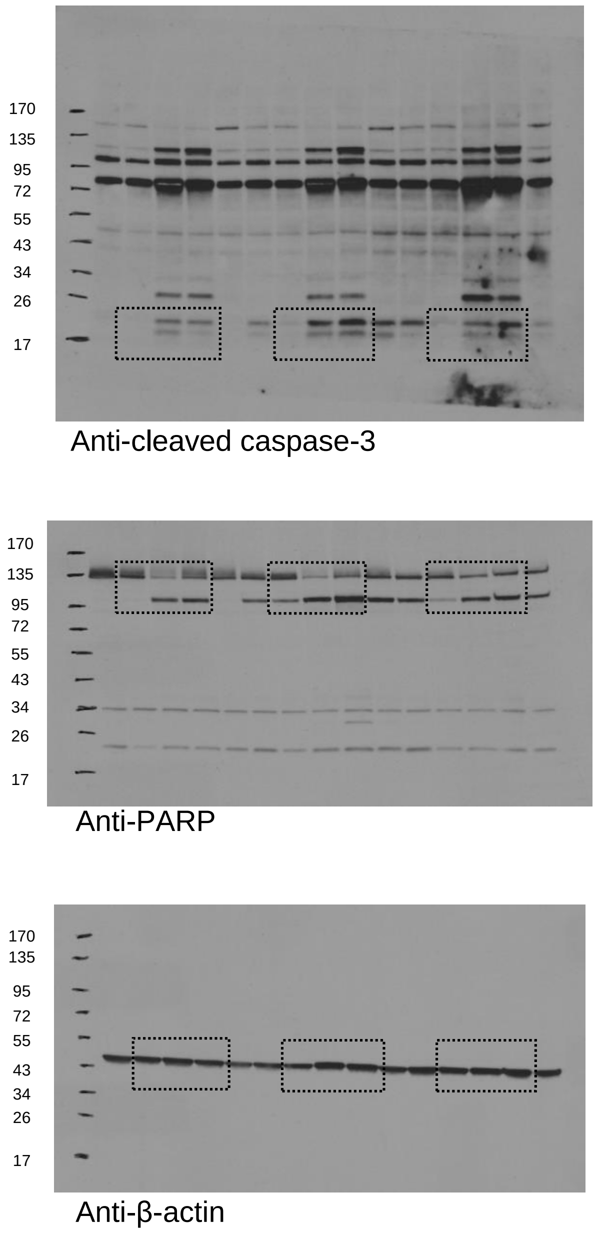

Supplemental Figure 4: Non-cropped western blot exposures from experiments using HCT116 cells. X-ray film exposures of western blots used in Figure 6C. Dash-outline box indicates areas used for figure.

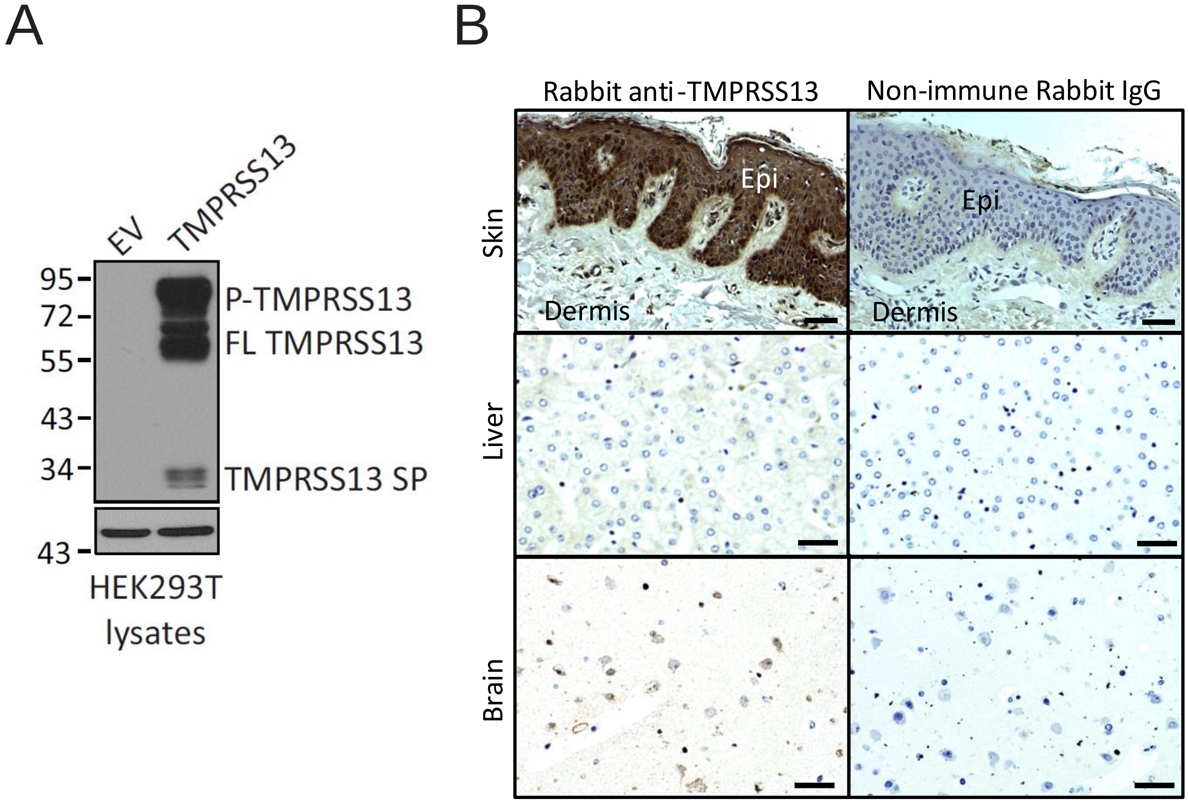

Supplementary Figure 5: Validation of anti-TMPRSS13 antibody used for IHC. A polyclonal antibody (PA5-30935, Thermo Fisher Scientific) that was raised against a recombinant protein fragment corresponding to a region within amino acids 195 and 562 of human TMPRSS13 was used for IHC analysis of human tissue. **(A)** Lysates from human HEK293T cells transfected with empty vector (EV) or S506A-TMPRSS13 were analyzed by reducing SDS-PAGE and western blotting using the PA5-30935 anti-TMPRSS13 antibody. No detectable bands were apparent in EV cells, whereas the full-length (FL) form and the phosphorylated (P) form of TMPRSS13 were readily detected in S506A-TMPRSS13 transfected cells in according with our previously published data (Murray et al., 2017). The proteolytically released serine protease (SP) domain of TMPRSS13 is also detected. **(B)** Representative samples from tissue array IHC analysis of TMPRSS13 protein in normal human tissue (human tissue arrays UNC241 and OR301, US Biomax Inc.). High expression of TMPRSS13 in human epidermis (Epi) (upper left panel) with low expression in the underlying dermis. The high expression level and pattern corresponds to previously published data using reporter mice with the *Tmprss13* gene disrupted by a β-galactosidase-neomycin fusion gene insertion (Madsen et al., 2014). Low expression of TMPRSS13 was observed in liver (middle panel) and brain (lower panel) corresponding to findings in mice (Madsen et al., 2014) and human tissue on the transcripts level (Kido et al., 2008). Primary, rabbit anti-TMPRSS13 antibody was substituted with non-immune rabbit IgG in serial sections (right panels) and no significant staining was observed. Scale bars=50 µm.

Murray, A. S. *et al.* Phosphorylation of the type II transmembrane serine protease, TMPRSS13, in hepatocyte growth factor activator inhibitor-1 and -2-mediated cell-surface localization. *The Journal of biological chemistry* **292**, 14867-14884, doi:10.1074/jbc.M117.775999 (2017).

Madsen, D. H., Szabo, R., Molinolo, A. A. & Bugge, T. H. TMPRSS13 deficiency impairs stratum corneum formation and epidermal barrier acquisition. *The Biochemical journal* **461**, 487-495, doi:10.1042/BJ20140337 (2014). Kido, H. & Okumura, Y. MSPL/TMPRSS13. *Frontiers in bioscience: a journal and virtual library* **13**, 754-758 (2008).

Kido H, Okumura Y, Takahashi E, Pan HY, Wang S, Chida J, Le TQ, Yano M. Host envelope glycoprotein processing proteases are indispensable for entry into human cells by seasonal and highly pathogenic avian influenza viruses. J Mol Genet Med. **29**;3(1):167-75 (2008).

A

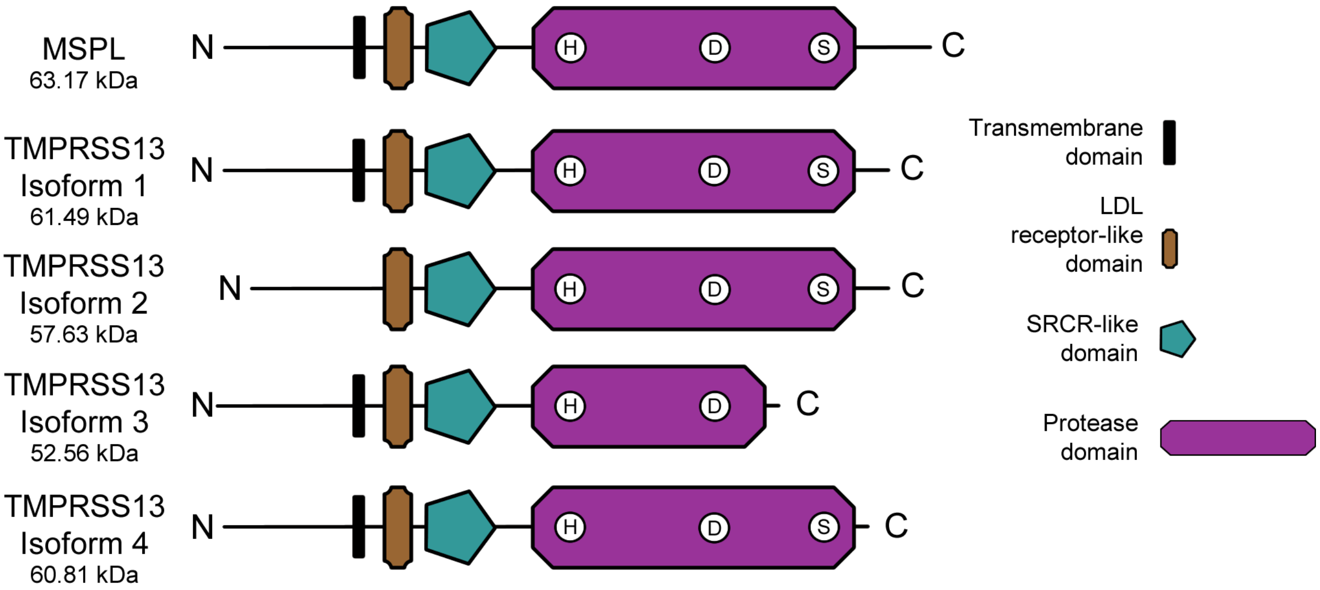

B

```
TMPRSS13_MSPL    144 RATRESPGTSLPKFTWREGQKQLPLIGCVLLLIALVVSLIILFQFWQ 190
TMPRSS13_iso 1   144 RATRESPGTSLPKFTWREGQKQLPLIGCVLLLIALVVSLIILFQFWQ 190
TMPRSS13_iso 2   144 RATRESP-----------------------------------VQFWQ 159
TMPRSS13_iso 3   144 RATRESPGTSLPKFTWREGQKQLPLIGCVLLLIALVVSLIILFQFWQ 190
TMPRSS13_iso 4   144 RATRESPGTSLPKFTWREGQKQLPLIGCVLLLIALVVSLIILFQFWQ 190
                     *******                                   .****

TMPRSS13_MSPL    421 SKPLTLSAHIHPACLPMHGQ---TFSLNET---CWITGFGKTRETD-----DKTSPFLRE  469
TMPRSS13_iso 1   421 SKPLTLSAHIHPACLPMHGQ---TFSLNET---CWITGFGKTRETD-----DKTSPFLRE  469
TMPRSS13_iso 2   386 SKPLTLSAHIHPACLPMHGQ---TFSLNET---CWITGFGKTRETD-----DKTSPFLRE  434
TMPRSS13_iso 3   421 SKPLTLSGEGI--CTPRSPAPQPQHPLQPSHLSASVNSYPGPKASAGQKSKTLKDPYMEH  478
TMPRSS13_iso 4   421 SKPLTLSAHIHPACLPMHGQ---TFSLNET---CWITGFGKTRETD-----DKTSPFLRE  469
                     *******..    * *        . *: :   . :..:   : :        ..*::..

TMPRSS13_MSPL    470 VQVNLIDFKKCNDYLVYDSYLTPRMMCAGDLRGGRDSCQGDSGGPLVCEQNNRWYLAGVT  529
TMPRSS13_iso 1   470 VQVNLIDFKKCNDYLVYDSYLTPRMMCAGDLRGGRDSCQGDSGGPLVCEQNNRWYLAGVT  529
TMPRSS13_iso 2   435 VQVNLIDFKKCNDYLVYDSYLTPRMMCAGDLRGGRDSCQGDSGGPLVCEQNNRWYLAGVT  494
TMPRSS13_iso 3   479 FCFII-----------------RETEAQG-L-----------------------------  491
TMPRSS13_iso 4   470 VQVNLIDFKKCNDYLVYDSYLTPRMMCAGDLRGGRDSCQGDSGGPLVCEQNNRWYLAGVT  529
                     . . :                  .  . * *

TMPRSS13_MSPL    530 SWGTGCGQRNKPGVYTKVTEVLPWIYSKMESEVRSLQQDTAPSRLGTSSGGDPGGAPRL 586
TMPRSS13_iso 1   530 SWGTGCGQRNKPGVYTKVTEVLPWIYSKMESEVRFRKS--------------------- 567
TMPRSS13_iso 2   495 SWGTGCGQRNKPGVYTKVTEVLPWIYSKMESEVRFRKS--------------------- 532
TMPRSS13_iso 3   491 ----------------------------------------------------------- 491
TMPRSS13_iso 4   530 SWGTGCGQRNKPGVYTKVTEVLPWIYSKMESSAG------------------------- 563
```

Supplementary Figure 6: TMPRSS13 is expressed as five isoforms. A) Visual representation of TMPRSS13 isoforms. Changes in protease domain and C-terminal protein length are shown. B) Sequence alignments for non-consensus regions among all five isoforms. Text color representative of corresponding domain represented in XA, demonstrating loss of TM sequence, truncated protease domain, loss of catalytic serine (highlighted in yellow), and changes in C-terminal sequence. * = fully conserved residue, : = strongly conserved residue, . = weakly conserved residue. Alignments performed using Clustal Omega alignment tool (Madeira et al., 2019).

Madeira F, Park YM, Lee J, Buso N, Gur T, Madhusoodanan N, Basutkar P, Tivey ARN, Potter SC, Finn RD nd Lopez R (2019) The EMBL-EBI search and sequence analysis tools APIs in 2019. *Nucleic acids research* **47**:W636-W641.

TMPRSS13 Isoform	Seq. Length (AA)	*RefSeq* / GenBank ID	Ensembl ID	TM	LDL receptor-like Domain	SRCR-like Domain	Protease Domain	Cataly-tic residues	Ref.
MSPL	586	Q9BYE2, BAB39741	ENST00000445164.6	YES	YES	YES	YES	H D S	(Kim et al., 2001)
Isoform 1	567	*NP_001070731.1* / AAI14929†, BAG62041	ENST00000524993.6	YES	YES	YES	YES	H D S	(Kido and Okumura, 2008)
Isoform 2, MSPS	532	*NP_001193718.1* / BAB39742	ENST00000528626.5	NO	YES	YES	YES	H D S	(Kim et al., 2001)
Isoform 3	491	*NP_001193719.1* / BAB55376	ENST00000526090.2	YES	YES	YES	PARTIAL	H D	(Ota et al., 2004)
Isoform 4	563	*NP_001231924.1* / AAO38062	ENST00000430170.6	YES	YES	YES	YES	H D S	(Clark et al., 2016)

Supplementary Table: Tabulated summary of reported isoforms of TMPRSS13. To date, five major isoforms of TMPRSS13 have been reported. The canonical form of TMPRSS13, isoform 1, contains four major ordered domains; major differences between isoforms with respect to these domains are summarized above. Ensembl IDs for the respective cDNA transcript are provided as well as RefSeq (in italics) and GenBank IDs for protein products. TMPRSS13 variation exists within isoforms, which can vary in the number of X-A-S-P-A-X amino acid repeats; protein products are reported to have between 11-13 repeats. Genbank IDs for polymorphic X-A-S-P-A-X repeat variants are provided alongside the respective canonical isoform. † = Sequence of TMPRSS13 isoform 1 used for overexpression experiments; this variant contains one less X-A-S-P-A-X repeat than the canonical sequence. MSPS = mosaic serine protease large-form, MSPS = mosaic serine protease short-form. TM=Transmembrane domain.

References

Clark K, Karsch-Mizrachi I, Lipman DJ, Ostell J and Sayers EW (2016) GenBank. *Nucleic acids research* **44**:D67-D72.

Kido H and Okumura Y (2008) MSPL/TMPRSS13. *Frontiers in bioscience : a journal and virtual library* **13**:754-758.

Kim DR, Sharmin S, Inoue M and Kido H (2001) Cloning and expression of novel mosaic serine proteases with and without a transmembrane domain from human lung. *Biochimica et Biophysica Acta - Gene Structure and Expression* **1518**:204-209.

Ota T, Suzuki Y, Nishikawa T, Otsuki T, Sugiyama T, Irie R, Wakamatsu A, Hayashi K, Sato H, Nagai K, Kimura K, Makita H, Sekine M, Obayashi M, Nishi T, Shibahara T, Tanaka T, Ishii S, Yamamoto J-i, Saito K, Kawai Y, Isono Y, Nakamura Y, Nagahari K, Murakami K, Yasuda T, Iwayanagi T, Wagatsuma M, Shiratori A, Sudo H, Hosoiri T, Kaku Y, Kodaira H, Kondo H, Sugawara M, Takahashi M, Kanda K, Yokoi T, Furuya T, Kikkawa E, Omura Y, Abe K, Kamihara K, Katsuta N, Sato K, Tanikawa M, Yamazaki M, Ninomiya K, Ishibashi T, Yamashita H, Murakawa K, Fujimori K, Tanai H, Kimata M, Watanabe M, Hiraoka S, Chiba Y, Ishida S, Ono Y, Takiguchi S, Watanabe S, Yosida M, Hotuta T, Kusano J, Kanehori K, Takahashi-Fujii A, Hara H, Tanase T-o, Nomura Y, Togiya S, Komai F, Hara R, Takeuchi K, Arita M, Imose N, Musashino K, Yuuki H, Oshima A, Sasaki N, Aotsuka S, Yoshikawa Y, Matsunawa H, Ichihara T, Shiohata N, Sano S, Moriya S, Momiyama H, Satoh N, Takami S, Terashima Y, Suzuki O, Nakagawa S, Senoh A, Mizoguchi H, Goto Y, Shimizu F, Wakebe H, Hishigaki H, Watanabe T, Sugiyama A, et al. (2004) Complete sequencing and characterization of 21,243 full-length human cDNAs. *Nature genetics* **36**:40-45.
